# Expression of hsa-miRNA-15b, -99b, -181a and Their Relationship to Angiogenesis in Renal Cell Carcinoma

**DOI:** 10.3390/biomedicines12071441

**Published:** 2024-06-27

**Authors:** József Király, Erzsébet Szabó, Petra Fodor, Anna Vass, Mahua Choudhury, Rudolf Gesztelyi, Csaba Szász, Tibor Flaskó, Nikoletta Dobos, Barbara Zsebik, Ákos József Steli, Gábor Halmos, Zsuzsanna Szabó

**Affiliations:** 1 Department of Biopharmacy, Faculty of Pharmacy, University of Debrecen, 4032 Debrecen, Hungary; kiraly.jozsef@pharm.unideb.hu (J.K.); fodor.petra@pharm.unideb.hu (P.F.); vass.anna@pharm.unideb.hu (A.V.); dobos.nikoletta@pharm.unideb.hu (N.D.); zsebik.barbara@pharm.unideb.hu (B.Z.); akos.steli@pharm.unideb.hu (Á.J.S.); halmos.gabor@pharm.unideb.hu (G.H.); 2 Doctoral School of Pharmaceutical Sciences, University of Debrecen, 4032 Debrecen, Hungary; 3 Department of Pharmacology, Faculty of Pharmacy, University of Debrecen, 4032 Debrecen, Hungary; erzsebet.szabo@med.unideb.hu; 4 HUN-REN-DE Pharmamodul Research Group, Department of Pharmaceutical Chemistry, Faculty of Pharmacy, University of Debrecen, 4032 Debrecen, Hungary; 5 Texas A&M Health Science Center, Department of Pharmaceutical Sciences, Irma Lerma Rangel School of Pharmacy, College Station, TX 77845, USA; mchoudhury@tamu.edu; 6 Department of Pharmacology and Pharmacotherapy, Faculty of Medicine, University of Debrecen, 4032 Debrecen, Hungary; gesztelyi.rudolf@pharm.unideb.hu; 7 Department of Pathology, Faculty of Medicine, University of Debrecen, 4032 Debrecen, Hungary; szasz.csaba@med.unideb.hu; 8 Department of Urology, Faculty of Medicine, University of Debrecen, 4032 Debrecen, Hungary; flash@med.unideb.hu

**Keywords:** kidney cancer, microRNA, hsa-miR-99b-5p, hsa-miR-15b-5p, hsa-miR-181a-5p, prognosis

## Abstract

Background: MicroRNAs (miRNAs) play a regulatory role in various human cancers. The roles of hsa-miR-15a-5p, hsa-miR-99b-5p, and hsa-miR-181a-5p have not been fully explored in the angiogenesis of renal cell carcinoma (RCC). Aims: The present study aimed to evaluate the expression of these miRNAs in tumorous and adjacent healthy tissues of RCC. Methods: Paired tumorous and adjacent normal kidney tissues from 20 patients were studied. The expression levels of hsa-miR-15b-5p, hsa-miR-99b-5p, and hsa-miR-181a-5p were quantified by TaqMan miRNA Assays. Putative targets were analyzed by qRT-PCR. Results: Significant downregulation of all three miRNAs investigated was observed in tumorous samples compared to adjacent normal kidney tissues. Spearman analysis showed a negative correlation between the expression levels of miRNAs and the pathological grades of the patients. Increased expression of vascular endothelial growth factor-A (VEGF-A) and hypoxia-inducible factor-1α (HIF-1α), a tissue inhibitor of metalloproteinases-1 (TIMP-1), was observed in tumorous samples compared to adjacent normal tissues. Depletion of tissue inhibitors of metalloproteinase-2 (TIMP-2) and metalloproteinase-2 (MMP-2) was detected compared to normal adjacent tissues. The examined miRNAs might function as contributing factors to renal carcinogenesis. However, more prospective studies are warranted to evaluate the potential role of miRNAs in RCC angiogenesis.

## 1. Introduction

Renal cell carcinoma (RCC) is the most common neoplasm of the kidney in adults, accounting for about 3% of all malignancies, and has the highest mortality rate of over 40% [1,2]. Over the past five decades, the incidence of RCC has rapidly increased, according to epidemiological data [3,4]. Clear cell RCC (ccRCC) is the most common type of RCC, which accounts for ~75–88% of all cases. Papillary RCC (pRCC) and chromophobe RCC (chRCC) are other common subtypes of renal cancer, with incidences of 6–15% and 2–5%, respectively [5,6]. At the time of diagnosis, metastases developed in approximately 20–30% of patients with RCC, and also, after curative surgery for localized RCC, another 30% of patients developed metastases in follow-up studies [3]. The current system used to predict the prognosis of patients diagnosed with renal cancer, while based on clinicopathological parameters, does not accurately predict the natural outcome of the disease, especially in localized primary RCC. Due to this failing prognostic system, there is an urgent need to find novel molecular biomarkers that can be used for the early diagnosis and evaluation of the prognosis of RCC and even serve as key therapeutic targets [2,7].

MicroRNAs are conserved, small (18~22 nucleotides), non-coding RNAs that play a major role in different molecular pathways by regulating gene expression [8,9]. In stable microvesicles, apoptotic bodies, or membrane-free carriers, miRNAs in human body fluids (blood plasma, urine, saliva, and semen) may have diagnostic biomarker roles and also indicate the prognosis of cancer diseases [10,11]. Based on the expression profiles, miRNAs can be indicators in distinguishing tumorous and normal tissues, even classifying tumors by histological type [10,11]. Aveta et al. [10], in a systematic summary, demonstrated that the expression panel of miRNAs is associated with a higher probability of diagnosing malignant renal masses, while other urinary miRNAs could be useful in distinguishing benign masses such as oncocytoma. According to the literature, some miRNAs, such as miR-3648, miR-489, miR-638, miR-3656, miR-3687, miR-25-5p, miR-21-5p, and miR-663b, have significant potential diagnostic value for ccRCC. Many of the identified miRNAs are associated with the regulation of the molecular signaling pathway involved in RCC tumor genesis. These miRNAs may serve as the basis for RCC therapy [11].

miRNAs have the ability to balance between pro- and antiangiogenic processes and are able to modify the appropriate course of events in angiogenesis [2]. For instance, some miRNAs (miR-23a, miR-21, and the miR-17-92 cluster) show pro-angiogenic properties, while others (miR-29b, miR-29c, and miR-192) prevent angiogenesis [8,9,12,13]. Earlier studies have described the role of miRNAs in tumor angiogenesis [13]. miR-210, which was identified as a hypoxia-regulated miRNA, was shown to have been upregulated in RCC [14]. Other studies indicated that miR-29b, a negative regulator of VEGF, also showed overexpression in RCC [14]. In recent studies, miR-126 has been shown to be correlated with the main angiogenic marker, VEGF-A [7]. This miRNA has also been linked to tumorigenesis in many types of cancer [4,7,14]. Various reports noted that VEGF-A receptors can be controlled by miR-200, which means this miRNA may also participate in angiogenesis [4,7,14]. An inverse correlation between miR-106a and miR-106b, the angiogenesis marker VEGF-A, was also found, which suggests a regulatory mechanism in this pathological condition [7]. Based on related studies, miRNAs represent potential therapeutic targets for the treatment of pathological neovascularization-related diseases due to their influence on multiple different pathways [15,16].

While previous studies have illustrated the role of miRNAs in renal cancer, many of their functions are not fully understood. Despite the large number of published studies that have followed, miRNAs in kidney tumors are not yet sufficiently known to be an effective tool in the diagnosis, prognosis, and therapeutic treatment of kidney cancer. In the present study, we compared the expressions of relevant RCC miRNAs, such as hsa-miR-15b-5p, hsa-miR-99b-5p, hsa-miR-181a-5p, and other angiogenesis-related genes, in both renal tumors and adjacent normal renal tissues from patients with RCC. In addition, our investigation sought to describe specific miRNAs and their roles in the progression of the angiogenesis process of renal cancer, as well as the potential prognosis of RCC. Utilizing statistical strategies, we correlated the expression patterns of the miRNAs of interest with clinicopathological parameters, including clinical stage and histology, to confirm our findings. Lastly, we aimed to describe the interactions of miRNAs and their targets as putative representatives of renal angiogenesis. Our long-term goal is to identify angiomiRs that may function in the angiogenesis pathway and could be used as potential targets for RCC therapy in the future.

## 2. Materials and Methods

### 2.1. Clinical Sample Collection

This study included patients admitted to the University of Debrecen, Department of Urology, for treatment of RCC between 2021 and 2023. Written informed consent was obtained from the medical Ethics Committee of the University of Debrecen (UD REC/IEC 4831-2017) prior to participation in the study. Tumorous specimens and adjacent normal kidney tissues were isolated from 20 patients with histologically proven kidney cancer who underwent surgical resection (mean age, 62 years; range, 46–78 years). From the collected cancer samples, all were diagnosed as primary tumors without any evidence of metastases. The clinicopathological data of the patients are shown in Table 1.

Tumors were staged using the TNM staging system of the Union for International Cancer Control, and histological grade was determined according to World Health Organization criteria [15]. Local invasion of the tumor cells was assessed using T staging, and lymphatic status was recorded as positive or negative. Due to the short investigation time period, follow-up with the patients was not considered. After the initial study metrics were collected, the tumor tissues were immediately frozen in liquid nitrogen and stored at −80 °C until further processing.

### 2.2. RNA Extraction and Quality Determination

Total RNA was extracted from tumorous kidney cancer and paired adjacent normal kidney tissues using the Qiagen RNeasy Mini isolation kit (Invitrogen, Life Technology, Carlsbad, CA, USA), according to the manufacturer’s protocol. RNA concentration and purity were determined by NanoDrop ND-1000 spectrophotometer (Nanodrop Technologies, Wilmington, DE, USA). An OD260/280 ratio of higher than 1.8 was assumed to be an indication of good RNA purity, thereby making it suitable for measuring gene expression. RNA integrity also was analyzed using the Agilent 2100 Bioanalyzer (Agilent Technologies, Santa Clara, CA, USA). Only RNA samples with 2.0 optical density at 260/230 nm were used for RT-PCR and further gene expression analyses. For further molecular biology analyses, RNA was stored at −80 °C.

### 2.3. TaqMan^®^ miRNA Quantitative Real-Time PCR and Statistical Analysis

qRT-PCR was performed using TaqMan^®^ microRNA assays (Life Technologies, Carlsbad, CA, USA). Complementary DNA (cDNA) for each miRNA of interest was synthesized from total RNA (5 ng) using the TaqMan^®^ microRNA Reverse Transcription Reagents (Invitrogen, Life Technology, Carlsbad, CA, USA) and specific reverse transcription primers (Life Technologies, Carlsbad, CA, USA). Real-time PCR was performed with TaqMan^®^ probes in a CFX-96 Real-time PCR System (Bio-Rad Laboratories Inc., Hercules, CA, USA) under the following conditions: denaturation at 95 °C for 10 min, followed by 40 repeating cycles of DNA polymerization at 95 °C for 30 s. The annealing step was performed at 60 °C for 1 min. All assays were performed in 96 well plates using triplicates. For each hsa-miR applied, the C_T_ values were determined using the SDS software (version 3.1) with automatic baseline and threshold settings. RNU6 was used to normalize each target miRNA. The data were loaded into the R statistical environment (v.2.14.0, Applied Biosystem, Foster City, CA, USA) and preprocessed. Triplicate C_T_ values were averaged and normalized to the geometric mean of RNU6, which was selected as an endogenous control based on geNorm27 and NormFinder (Department of Molecular Medicine (MOMA), Aarhus University Hospital, Aarhus N, Denmark). The normalized expression was calculated as log2|2^−ΔCT^|. C_T_ values > 36 were considered to be below the limit of detection.

### 2.4. In Silico miRNA Analysis for Target and Pathway Prediction

In the case of hsa-miR-15b-5p, hsa-miR-99b-5p, and hsa-miR-181a-5p chosen for the study, based on literature data, an in silico study was carried out by comparing miRNA-specific targets with the help of 3 databases: miRDB, TargetScan, and Tarbase. A search for angiogenesis signaling pathway-related target proteins was the main purpose of this process. Based on the database screening results, we determined the miRNA-target interactions and performed enrichment analysis.

### 2.5. Tissue Lysate Preparation for Protein Array Analysis

Proteins from 8-8 adjacent tumorous and healthy renal cancer tissue samples were extracted with Lysis buffer 6 or Lysis buffer 17 (provided in the Proteome profiler Human Angiogenesis Array Kit (ARY007, Bio-Techne, McKinley, MN, USA). After incubating and rocking at 4 °C for 30 min, the samples were centrifuged at 14,000× *g* for 5 min, supernates were transferred into clean tubes and subjected to protein quantification. The total protein content of the samples was quantified by the Bradford Method. During isolation and quantification, samples were kept on ice to avoid degradation and stored at −80 °C.

For each membrane supplied in the kit, 300 µg of protein extract was used for Human Angiogenesis Array analysis. Proteins were mixed with a Detection Antibody Cocktail (supplied) and incubated at room temperature for one hour. The sample/antibody mixture was then added to the array membranes and incubated overnight at 4 °C. The next day, all of the membranes were washed with 1x Wash Buffer (supplied) three times, then incubated with Streptavidin-HRP at room temperature for 30 min on a rocking platform shaker. Following incubation, the arrays were washed three times with a washing buffer, and the signal of the protein spots was visualized by chemiluminescence measurement using the reagent supplied in the kit. ChemiDoc Imaging System (Bio-Rad, Hercules, CA, USA) was used for the membrane visualization and for the quantification of band density. The intensity score of each duplicated array spot was measured with the Image Lab software (version 5.2.1., Bio-Rad, Hercules, CA, USA), and the average intensity was calculated by subtracting the average background signal. The fold change was obtained by comparing the tumorous tissue samples with the normal samples (indicated as a value of 1). The identity and the respective coordinates of all antibodies on the arrays can be found in the “Supplementary Material” provided in the kit (ARY007).

### 2.6. Reverse Transcription PCR (RT-PCR)

cDNA was synthesized from 250 ng of RNA from each sample using the Tetro cDNA Synthesis Kit (Bioline, London, UK) according to the manufacturer’s guidelines. The RT-PCR reaction was performed in a 20 μL volume using random hexamers (Table S1). The run consisted of 35 cycles (95 °C for 15 s, 60 °C for 30 s, 72 °C for 10 s, and 72 °C for two minutes). To test for contamination, RT-NTC was incorporated into the reaction.

### 2.7. Quantitative Real-Time PCR (qRT-PCR)

qRT-PCR was conducted using the iTaqTM Universal SYBR^®^ Green Supermix (Bio-Rad Laboratories Inc., Hercules, CA, USA). The reaction was performed in a CFX-96 Real-Time System (Bio-Rad Laboratories Inc., Hercules, CA, USA) in a final volume of 20 µL. 10 min of preheating at 95 °C was followed by 45 cycles at 95 °C for 15 s and 60–65 °C for 1 min. The oligo sequences and the primer-specific annealing temperatures used for the real-time qPCR are listed in Table S3 (each reaction was performed in triplicate). Following this, the relative value of mRNA was determined by the C_T_ technique using threshold cycle times for each mRNA. Triplicate C_T_ values were averaged and normalized to the average Cp mean of GAPDH, which were selected as endogenous control. The normalized expression was calculated as log2|2^−ΔCT^|. 

### 2.8. Statistical Analysis

#### 2.8.1. Statistical Analysis of the Expression of hsa-miRNAs in Tumorous and Adjacent Healthy Kidney Cancer Tissues

Using the TaqMan miRNA assay (Life Technology, Carlsbad, CA, USA), hsa-miR-15b-5p, hsa-miR-99b-5p, and hsa-miR-181a-5p expression levels were quantified. For statistical analysis, two-way ANOVA with Sidak multiple comparison tests were used based on GraphPad Prism 9.5.1 for Windows (GraphPad Software Inc., La Jolla, CA, USA).

#### 2.8.2. Statistical Analysis of the Correlation of the miRNAs Expression Level with Pathological Grades of the Patients

The relationship between the miRNA expression and the pathological grade of the patients was investigated in three ways, i.e., with and without identifying outliers (in the last case, using the ROUT method with two different levels of “aggressiveness”, Q = 1% and Q = 5%) in the miRNA expression data. For each dataset, the correlation coefficient (r) between variables (expression and grade) was calculated using Spearman’s (nonparametric) method. The precision of the correlation was characterized by a 95% CI of r. If the correlation was found to be statistically significant, linear regression was performed, and its precision was visualized by the 95% confidence bands around the related best-fit straight line.

The *p* value less than 0.05 was considered to be statistically significant. The levels of significant differences were the following: *p* < 0.05 (*), *p* < 0.0021 (**), *p* < 0.0002 (***), and *p* < 0.0001 (****). All statistical analyses were performed with GraphPad Prism 9.5.1 for Windows (GraphPad Software Inc., La Jolla, CA, USA) and Microsoft Excel 365 (Microsoft Co., Redmond, WA, USA).

## 3. Results

### 3.1. Clinicopathological Characteristics of the Patients

A histopathological examination of each specimen (N = 20) was performed to confirm the presence of cancer with minimal mixed nonmalignant tissue. According to the pathologist’s overview, the samples used in the study were classified as clear cell renal cell carcinoma (ccRCC): 16 cases (78%), papillary type: 2 cases (10%), and chromophobe type: 2 cases (10%). Within a histological type, all the tissues were analyzed according to Fuhrman Grades: 62.5% (13 samples) of the samples were classified as Grade 2 (low-grade), 20% (4 samples) as Grade 1 (low-grade), and 15% (3 samples) as Grade 3 (high-grade). Eight (40%) of the examined kidney tissue samples were isolated from male patients, and 12 samples (60%) originated from female patients (Table 1).

### 3.2. Angiogenesis-Related miRNAs Expression

**Figure 1:**
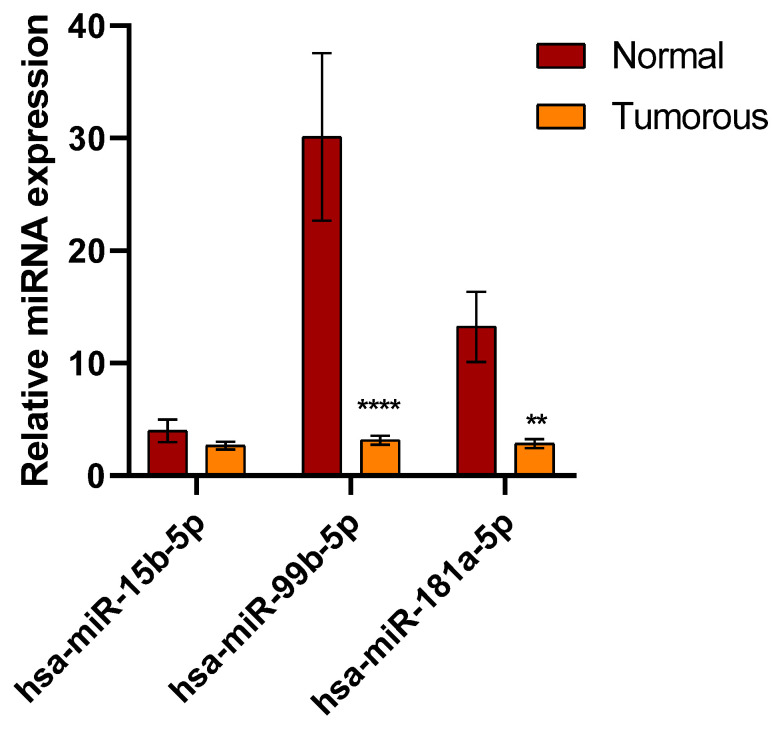
Analysis of miRNA expression in tumorous and paired healthy renal cancer tissue samples by TaqMan assays. RNU6 was used as an endogenous control miRNA to normalize each target miRNA. Two-way ANOVA with Sidak multiple comparison test was used for statistical analysis (** *p* = 0.0090, **** *p* < 0.0001).

To explore whether the miRNAs were affected during the progression of kidney cancer, hsa-hsa-miR-15b-3p, hsa-miR-99b-5p, and miR-181a-5p expression was analyzed in cancerous and paired healthy renal tissue samples by TaqMan RT-qPCR. As shown in Figure 1, all of the three miRNAs examined were downregulated in tumorous tissues compared to adjacent normal healthy tissues.

A significant difference (*p* ≤ 0.05) was found in the expression levels of miRNAs between tumorous and paired healthy tissues of patients with RCC (Figure 1). The most significant differences were observed in the expression of hsa-miR-99b-5p (*p* < 0.0001) and hsa-miR-180a-5p (*p* = 0.0090) between the healthy and tumorous samples (Figure 1).

### 3.3. Correlation of Patients’ miRNAs and Tumor Stages

The patients were included in different pathological stages (grades 1, 2, and 3) of RCC. Thus, the expression of the studied miRNAs in tumorous and adjacent normal tissues was analyzed according to the pathological status of the patients.

**Figure 2:**
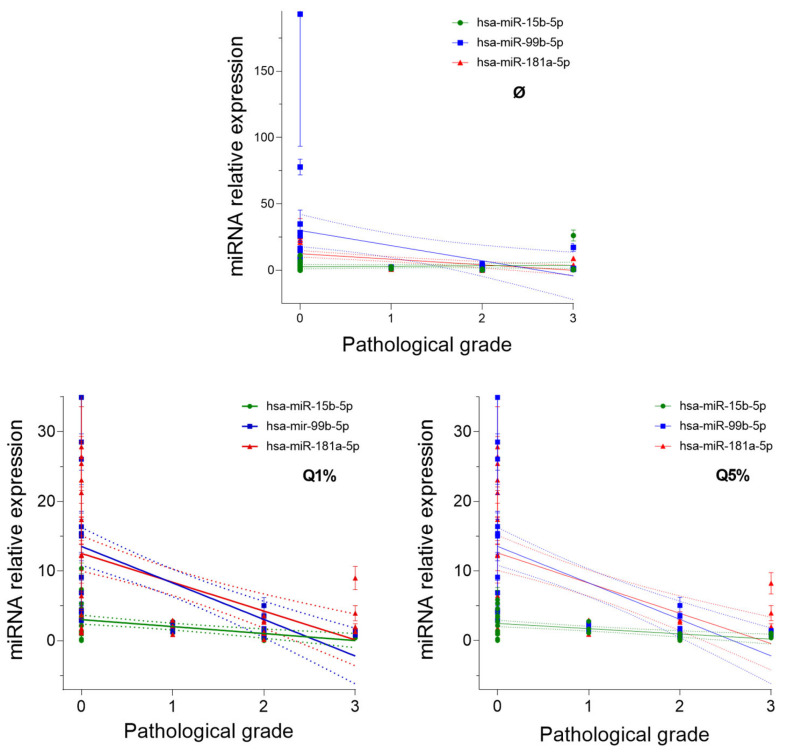
The relative expression of three types of microRNAs (hsa-miR-15b-5p, hsa-miR-99b-5p, and hsa-miR-181a-5p) against the pathological grade of the holding tissue, without identifying outliers (Ø), identifying outliers with Q = 1% (Q1%), and identifying outliers with Q = 5% (Q5%). The symbols represent the mean (±SEM) of three technical replicates (subtracting the identified outliers, where appropriate).

For all three miRNA types in all cases, we found a statistically significant negative correlation between relative expression and pathological grade of the holding tissue. The correlation and the statistical significance were the strongest, and the determination was the most precise when Q = 1% (medium “aggressiveness”) was chosen for the outlier identification (Table S2). The results obtained by linear regression matched the results of the correlation analysis (Figure 2). According to Spearman correlation, *p* values were determined for each miRNA examined as follows: *p* = 0.033 for hsa-miR-15a-5p, *p* < 0.0001 for hsa-miR-99b-5p, and *p* = 0.0133 for hsa-miR-181a-5p (Figure 2).

The expression of the miRNAs examined was significantly lower in all three pathological grades. The most significant difference between expression levels was observed in grades 2 and 3 tissues (*p* ≤ 0.05). There was an approximately three-fold decrease in the expression of all miRNAs examined in tumorous samples compared to adjacent healthy tissues in Grade 3. However, only hsa-miR-99b-5p and hsa-miR-15b-5p showed a three-fold decrease in grades 1 and 2. Regarding pathological grades, hsa-miR-99b-5p and hsa-miR-181a-5p showed higher expression in tumorous samples (Table S2, Figure 2).

Significant differences were observed in the expression of hsa-miR-99b-5p (*p* < 0.0001) and hsa-miR-181a-5p (*p* = 0.0090) between the healthy and tumorous samples.

### 3.4. Correlation of Patients’ miRNAs and Lymph Node

In total, most of the patients had negative lymph node status as metastasis was not detected. Only in one of the patients, regional lymph node metastases was described (Table 1). Therefore, the correlation between the expression of hsa-miR-15b-5p, hsa-miR-99b-5p, and hsa-miR-181a-5p and lymph node status was not observed.

### 3.5. In Silico miRNA Target Database Analysis

**Figure 3:**
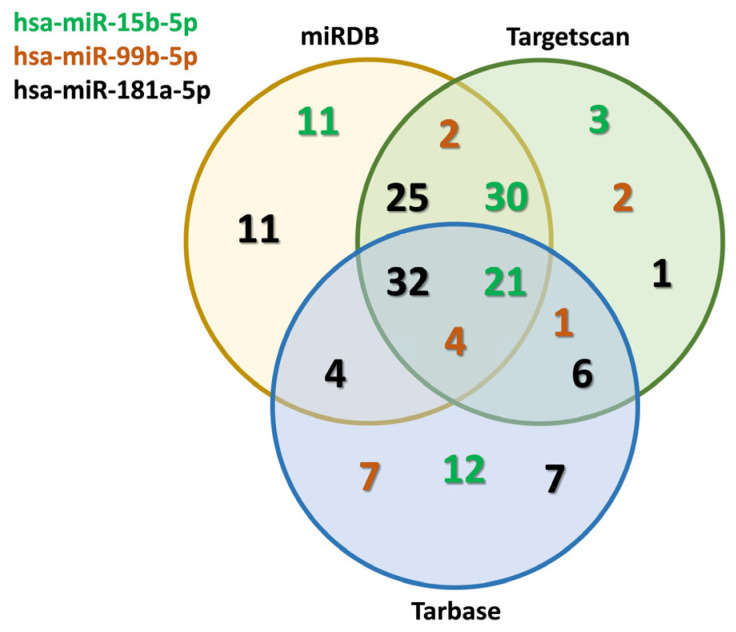
TaqMan assays validated miRNAs and their putative targets in angiogenesis. Screening three different databases, the potential targets for each miRNA were identified. The Venn chart shows the identified targets of the miRNAs in one database, and the common targets in all three databases are visualized by numbers. The targets that can be found in all three databases are listed in Table S3A.

In silico analyses of three distinct miRNA databases were performed in search of the angiogenesis pathway-specific targets on three different databases, including miRDB (https://mirdb.org/, accessed on 2 November 2023), Tarbase (https://dianalab.e-ce.uth.gr/tarbasev9/interactions, accessed on 7 November 2023) and TargetScan (https://www.targetscan.org/vert_80/, accessed on 9 November 2023). Screening these databases for hsa-miR-15b-5p, hsa-miR-99b-5p and hsa-miR-181a-5p, the potential targets for each miRNAs were identified. The targets found in all three databases are highlighted in Table S3A. The Venn chart (Figure 3) shows the most common angiogenesis pathway-related targets of miRNAs found in the three databases. By numerically plotting the targets, the overlaps across the sets revealed a number of targets that could be involved in the process of angiogenesis, including Fibroblast growth factor 1 (FGF-1), Vascular Endothelial Growth Factor-A (VEGF-A), Serpin peptidase inhibitor clade A1 (SERPIN-A1), Matrix metalloproteinases (MMPs), Hypoxia-inducible factor 1-alpha (HIF-1α), Tissue inhibitors of metalloproteinases (TIMPs). The whole list of the targets is found in the Table S3B. The common targets among two or all three of the databases are represented by numbers found in Figure 3. According to database analyses for hsa-miR-181a-5p, we found 32 common targets, including VEGF, TIMPs, and MMPs.

Similarly, for hsa-miR-15b-5p, our search identified 21 validated targets, among which VEGF, FGF-1, and FGFR1 were found to be relevant. In addition, VEGF and TIMPs were found to be commonly validated for hsa-miR-99b-5p (Figure 3). These database analyses revealed that all three studied miRNAs could be involved as epigenetic factors in the pathological process of renal cancer angiogenesis [10].

### 3.6. Evaluation of the Angiogenesis Array

There are a number of molecular markers involved in the process of angiogenesis that lead to the development of kidney cancer. The purpose of this study was to reveal the connection between major angiogenesis factors and related miRNAs. We used a proteome profile analysis to screen for the most dominant angiogenic markers in the primary cancer tissue samples compared to adjacent healthy tissues. A Human Angiogenesis Array (Bio-Techne, ARY007, McKinley, MN, USA) was also employed to study early signaling events.

**Figure 4:**
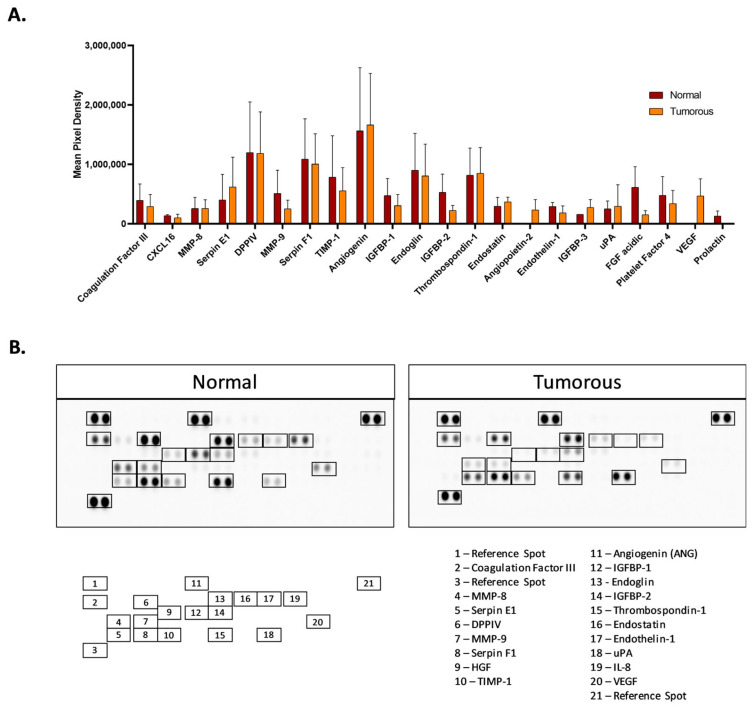
Protein expression analyses on normal and tumorous human renal tissues using the Human Angiogenesis Array Kit. Array spots were visualized in accordance with the manufacturer’s instructions. The intensity of each spot was measured with the ChemiDoc Imaging System (Bio-Rad, Hercules, CA, USA). The data show the mean intensity of eight normal and eight tumorous sets of samples (**A**) and the location of each protein in a representative membrane of tumorous and adjacent normal (healthy) tissue samples (**B**).

A Human Angiogenesis Array has the ability to detect angiogenesis biomarkers in the protein lysate of tumorous and adjacent healthy kidney cancer tissues. After chemiluminescence detection, the spot intensity showed the expression of specific proteins placed on the array. Based on the results obtained from eight healthy and eight tumorous kidney tissues, the average spot density was analyzed and calculated using the Chemidoc Image Analyser (Bio-Rad, Hercules, CA, USA). The combined results from all the 8-8 paired tumorous and healthy kidney cancer samples are shown in the bar chart. A representative analysis performed on one set of the tumorous and adjacent healthy tissues can be seen in Figure 4A,B. (Figure S1 shows the membranes of each sample.)

The evaluation of angiogenesis arrays showed an increase in the expression of angiogenic proteins, such as ANG, in tumorous samples, while a slight decrease in the expression of MMP-9 was observed during the screening. In tumorous tissues, TIMP-1 also showed a slight decrease compared to healthy samples. Based on the protein array analyses, most likely one of the main angiogenic markers, VEGF, has a very low expression level (not even visible) in healthy tissues. However, in tumorous samples, we could observe significant amount of VEGF.

### 3.7. Results of Real-Time qRT-PCR

To ensure that the altered changes in Human Angiogenesis arrays were due to upstream or downstream regulation for validating specific gene expression at the mRNA level, we performed sequence-specific primer-based qRT-PCR analyses. All the 20 paired healthy and tumorous kidney samples were analyzed with the help of gene-specific primers for VEGF-A, FGF-1, ANG, MMP-9, and TIMP-1. The expression of HIF-1α, MMP-2, and TIMP-2 also was analyzed by real-time qRT-PCR. As indicators of both physiological and pathological conditions of angiogenesis, vascular endothelial growth factor receptor 1 (VEGFR-1) and vascular endothelial growth factor receptor 2 (VEGFR-2) were also measured.

**Figure 5:**
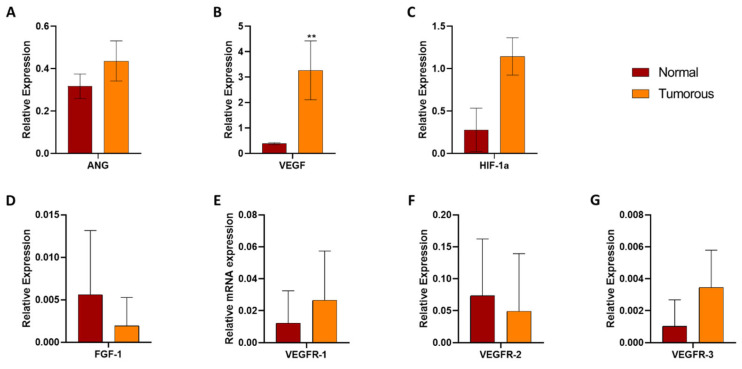
Angiogenesis-related gene expression in tumorous and adjacent kidney cancer tissues studied: ANG (**A**), VEGF (**B**), HIF-1α (**C**), and FGF-1/VEGFR-1-2-3 (**D**–**G**) analyses in human renal tissues. All qRT-PCR experiments were performed in triplicate. GAPDH was used as a housekeeping gene. Two-way ANOVA with the Sidak multiple comparison test was used for statistical analysis. VEGF (**B**) showed a significant difference in expression (** *p* = 0.0011). The sequences of primers are listed in Table S4.

**Figure 6:**
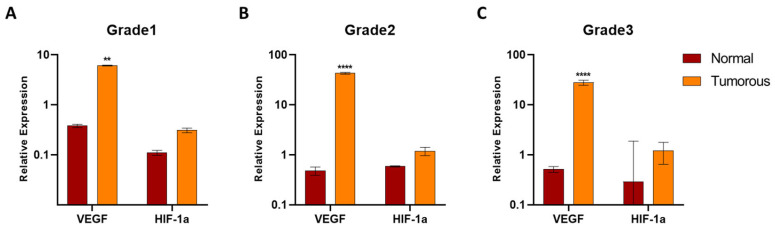
VEGF and HIF-1α expression in studied tumorous and adjacent kidney cancer tissues, represented by pathological grades. Two-way ANOVA with the Sidak multiple comparison test was used to detect significant differences. The change in the expression of VEGF was significant in all three pathological grades: in Grade 1 (**A**) ** *p* = 0.0014, in Grade 2 (**B**) and in Grade 3 (**C**) **** *p* < 0.0001.

ANG, VEGF, and HIF-1α were significantly upregulated (*p* ≤ 0.05) in the tumorous samples and significantly downregulated (*p* ≤ 0.05) in paired healthy tissues (Figure 5 and Figure 6). This slight discrepancy in the results obtained by qRT-PCR and the angiogenesis array may be a result of the low representative number of the samples used for screening the main angiogenesis markers in RCC samples.

The expression of FGF-1 and VEGFRs was also investigated because both of them participate in the angiogenesis of RCC. Real-time PCR analysis verified the results of the Human Angiogenesis Array. The downregulation of FGF-1 was shown in tumorous tissues, and the expression of VEGFR-1, -2, and -3 receptors was also detected both in tumorous and adjacent healthy tissues of RCC (Figure 5).

Additionally, observing results in the light of the pathological grades of the samples. RCC samples showed higher HIF-1α expression than samples identified with lower grades. Intensive VEGF expression in tumor cells was observed in low-grade (Grade 1 and 2) RCC samples and in high-grade (Grade 3) RCC samples as well (Figure 6).

**Figure 7:**
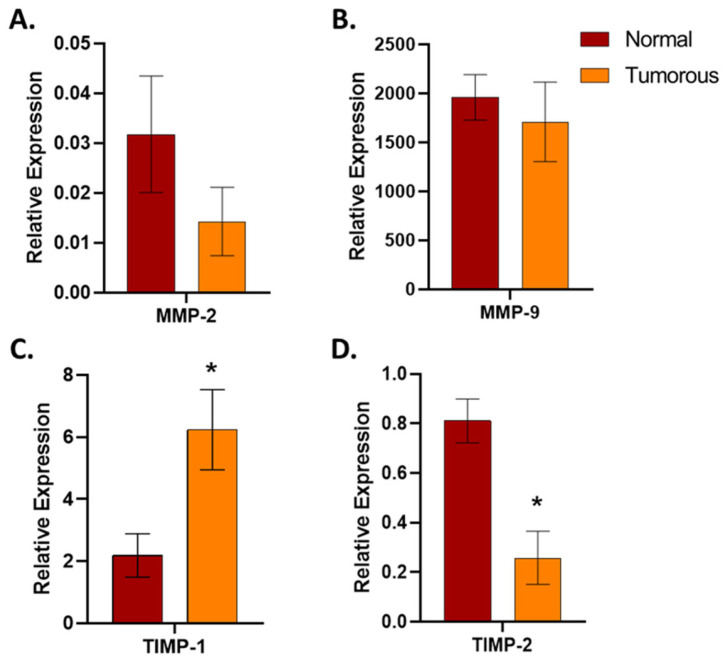
MMP and TIMP expression analysis in human renal tissues. All qRT-PCR experiments were performed in triplicates. GAPDH was used as the housekeeping gene. Two-way ANOVA with Sidak multiple comparison test was used for statistical analysis. MMP-2 (**A**) and MMP-9 (**B**) did not show significant differences (*p* > 0.9999); however, the difference in the expression of TIMP-1 (* *p* = 0.0328) and TIMP-2 (* *p* = 0.0371) was significant (**C**,**D**).

Regarding the expression of MMPs and TIMPs, the quantitative RT-PCR results were mostly consistent with the results of the Human Angiogenesis Array. The results of gene expression analysis demonstrated a slight decrease in the expression of MMP-9 and an increase in the expression of TIMP-1 (Figure 7) in tumorous samples compared to normal adjacent tissues, which correlates with an increase in the expression of these proteins. We also analyzed the expression of MMP-2 and TIMP-2 in the samples of the study. Both genes showed lower expression in tumorous tissues than in the adjacent healthy kidney tissue samples (Figure 7).

## 4. Discussion

Angiogenesis is a complex process involving the synchronous activity of diverse pro- and anti-angiogenic factors [5,16,17,18]. It also plays a vital role in the development of RCC and its histotypes [16,19,20]. The control mechanisms of angiogenesis in carcinogenesis are tightly regulated at the genetic and epigenetic levels [16]. Gene silencing in cancer cells is mainly based on genetic variation. Epigenetic dysregulation can downregulate tumor suppressor genes or oncogene activation, playing an important role in tumor development in both the early and late stages [16,19,20]. The whole regulatory mechanism of angiogenesis in RCC has not yet been extensively explored. The identification of dysregulated angiogenesis-associated genes, specific angiogenesis-related markers, and their regulating miRNAs is crucial for understanding their role in the regulation of the whole process in kidney cancer [18,19]. The miRNA expression profile of RCC determines new prognostic factors and serves as an explanation for the molecular mechanisms involved in disease development and progression [2,7]. Many of the main genes involved in the process of angiogenesis may be controlled by the activity of one or more miRNAs [3]. Although a significant number of miRNAs have already been described in the literature, the association between miRNA expression profiles and the pathological status or prognosis of RCC has not yet been completely demonstrated [2,7,13].

The roles of hsa-miR-15b-5p, hsa-miR-99b-5p, and hsa-miR-181a-5p in the process of angiogenesis also have not yet been fully elucidated. Our results that studied 20 kidney cancer and adjacent healthy control tissues clearly demonstrated that in all the samples examined, the miRNAs of interest were significantly downregulated in tumorous tissues compared to healthy adjacent pairs. A negative correlation between the expression level of miRNAs and the pathological grades of the patients was also supported by linear regression analysis. Overall, each of the miRNAs examined showed low expression in all tissue grades (grades 1, 2, and 3). In this regard, our results were consistent with studies of Redova et al., where TaqMan low-density arrays were used to identify differentially expressed miRNAs in tumorous and adjacent normal tissues of patients diagnosed with ccRCC: 73 miRNAs were downregulated, and 5 miRNAs were upregulated in tumors [13]. Tumorous and adjacent normal tissues of patients with ccRCC have shown that miRNAs, like miR-221, miR-222, miR-130a, let-7f-1, miR-27b, miR-378, miR-210, miR-15a, miR-16-1, and miR-126 are involved in angiogenesis [4,7].

Looking at each of the miRNAs tested separately, we found significant downregulation of hsa-miR-15b-5p. These findings are consistent with a report made by Kumar et al., 2022 in which the tissues of patients diagnosed with non-small cell lung cancer (NSCLC) were observed to have a significant downregulation of miR-15a-5p [21]. Kao et al., 2017 demonstrated that miR-15a directly regulate the expression of Programmed Cell Death-Ligand 1 (PD-L1) in malignant pleural mesothelioma [22]. Other reports also described the tumor suppressor role of miR-15a [21,22,23,24]. Kumar et al. 2022 showed that extracellular vesicles overexpressing miR-15a inhibited the immune evasion of colorectal cancer cells via the Lysine-specific demethylase 4B/Homeobox protein Hox-C4/Programmed death-ligand (KDM4B/HOXC4/PD-L1) axis [21].

In our study of tumorous kidney tissue samples, hsa-miR-99b-5p was also significantly downregulated compared to healthy adjacent paired samples. These results are consistent with the findings of Cui et al. (2012), who reported that miR-99a was remarkably downregulated in RCC, resulting in a low expression level of miR-99a, which correlated with poor survival of patients with RCC [4,7].

Hsa-miR-181a-5p has previously been shown to play a vital role in cancers [25]. In the current study, analysis of hsa-miR-181a-5p showed a clear downregulation in adjacent tumorous samples, which is consistent with previous studies that demonstrated the downregulation of miR-181a-5p in aggressive human breast and colon cancers, whereas its expression level was inversely correlated with MMP-14 expression level [25,26].

The study of Yulin Lai et al. [25] demonstrated that miR-181a-5p is upregulated in RCC tissues and cell lines and is associated with cell migration, proliferation, and apoptosis in RCC. Thus, miR-181a-5p may function as an oncogene or tumor suppressor via one or more signaling pathways in certain types of tumors [25,26].

**Figure 8:**
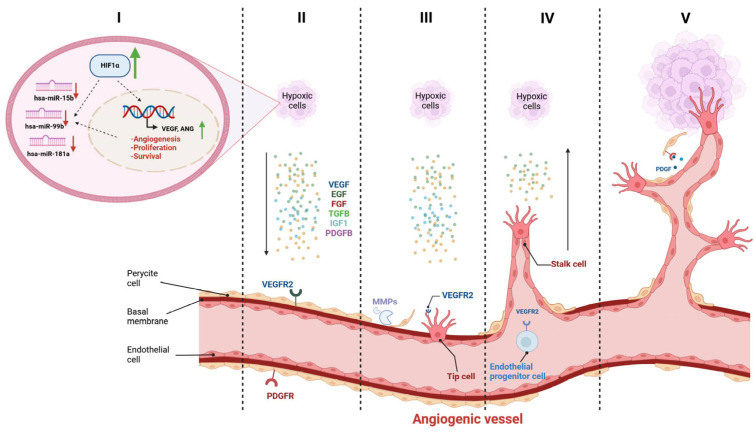
Regulation of angiogenesis. Under the influence of angiogenic stimuli, new blood vessels develop from the pre-existing vessels. In tumors, the reduced oxygen concentration induces the accumulation of HIF, which leads to the increased expression of VEGF and other angiogenic factors. Possibly, this hypoxic environment results in the downregulation of the miRNAs we tested (I). Increased expression of pro-angiogenic factors, including VEGF-A, stimulates VEGFR-2 receptors on endothelial cells in the blood vessels (II). This leads to the detachment of pericytes from the basal membrane, and a tip cell is selected to guide elongation (III). Stalk cells proliferate and elongate toward hypoxic cells (IV), and the new vessel is stabilized by pericytes by PDGFR (V) [1].

Angiogenesis is a multistage process where new blood vessels develop from pre-existing vessels as a result of an angiogenic stimulus [5,25]. In addition, the reduced oxygen concentration induces the accumulation of HIF and leads to the increased expression of VEGF. Accumulation of HIF, VEGF, PDGF, and FGF is associated with increased angiogenesis and the metastatic potential of RCC [4,7,16,18,27,28]. Upon binding to its receptors, VEGFR-1, VEGFR-2, and VEGFR-3, VEGF-A mediates intracellular signaling mechanisms in ECs. VEGFR-2 has been mainly attributed to angiogenesis, while VEGFR-1 inhibits angiogenic processes by maintaining VEGFR-2 levels. VEGFR-3, a receptor for VEGF-C, is involved in vascularization in the early embryonic phase. (Figure 8) [2]. The expression of all three receptors in analyzed samples is one of the results that may underline the ongoing angiogenesis [2,12,29].

The expression of VEGF also promotes the dysregulation in the expression of further angiogenesis-related genes, such as MMPs and their inhibitor proteins, TIMP-1 and TIMP-2. VHL/HIF1 signaling leads to increased levels of cyclic adenosine monophosphate (cAMP) response element binding protein (CREB), a transcription factor that upregulates the expression of pro-angiogenic miRNAs by promoting the expression of VEGF [2,4,12,29,30,31,32,33].

**Figure 9:**
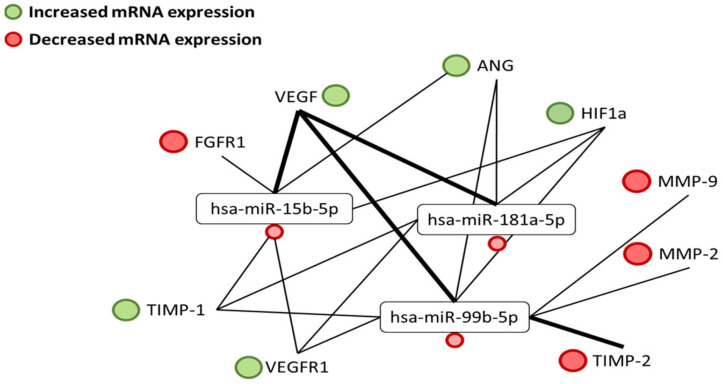
Interactions between miRNAs and angiogenetic targets in RCC specimens. The connecting lines show the interactions between the studied miRNAs and hypothetical putative targets. The thicker lines show the common targets found in all three databases.

In silico analysis of three distinct miRNA databases clearly revealed common targets that might play a role in angiogenesis: HIF-1α, VEGF, FGF-1, MMPs, and TIMPs [34] (Figure 9). In our study, the expression of these targets was screened at the protein level and analyzed at the mRNA level. Reduced oxygen concentration primarily induces the accumulation of HIF1-α and leads to the increased expression of VEGF-A [2,29,31,34]. In the human RCC specimens investigated, we observed a significant increase in the expression of VEGF-A in tumorous tissues compared to adjacent healthy tissue samples. This increase correlates with the increased expression of related proteins, like ANG, which also intensively contributes to kidney cancer angiogenesis [35]. Based on these results, we postulate that in the samples studied, the initial steps of angiogenesis would not particularly be controlled by miRNAs, but rather, the hypoxic environment induces the expression of the tested miRNAs. We assume that through miRNA-target interaction, the increased VEGF-A and HIF-1α levels directly regulate the expression of the miRNAs analyzed. This increase could be the main reason behind miRNA downregulation [26]. The downregulated miRNAs, hsa-miR-15b-5p, hsa-miR-99b-5p, and hsa-miR-181a-5p, through specific miRNA-target interactions may later take over the role of epigenetic regulation of angiogenesis in early primary tumors (Figure 9). We also hypothesize that the tested miRNAs possibly function as angiomiRs, promoting the progression of angiogenesis through the formation of new vessels [4,8,23]. Most likely, a specific interaction of all three studied miRNAs with potent inducers of angiogenesis, such as ANG and VEGF, might lead to the development of new blood vessels. They interact with endothelial and smooth muscle cells, which allow them to infiltrate, proliferate, and form tubular structures (Figure 8 and Figure 9) [2,29,32,33,34,36]. Considering the expression pattern of VEGF in the examined samples, we may assert that mainly the upregulated VEGF could be a key regulator in the formation of new blood vessels and downregulation of miRNAs [2,29]. According to Chun-Yan Sun [9], hsa-miR-15a acts as a putative tumor suppressor by targeting the oncogene B-cell lymphoma (BCL-2), which has been implicated in apoptosis and proliferation. It was shown that ectopic overexpression of miR-15a led to decreased pro-angiogenic activity of cancer cells, and miR-15a plays a substantial role in the tumorigenesis, at least in part, by the modulation of angiogenesis through targeting VEGF-A [9]. The relationship between the expression of Programmed cell death 1 ligand 1 (PD-L1) and VEGF, MMP-9, and KI-67 was studied earlier in glioma [8,23]. The expression rate of PD-L1 positively correlated with the tumor grade and VEGF status, suggesting that PD-L1 has a function in angiogenesis and proliferation [24]. Many other studies have reported that lower expression of miR-15a correlates with poor prognosis in many types of cancer [21]. It has also been demonstrated that the deregulation of miR-99a is involved to a certain extent in the biology of RCC by directly targeting the mTOR pathway. This suggests that miR-99a could be a promising target for diagnostic and therapeutic purposes in RCC [4,23,24,27].

Hypoxia-inducible factor-1α, another key player in vasculature formation, upregulates other pro-angiogenic factors, which showed a higher expression in tumorous tissues compared to normal adjacent tissue samples in our study. We can assume that the miRNAs examined contribute to the sprouting process of angiogenesis, and additionally, the expression of these miRNAs would lead to increased expression of VEGF and HIF-1α [8,34]. Nevertheless, hypoxia plays a key role in angiogenesis by decreasing the degradation of the transcription factor HIF-1α by ubiquitination, resulting in the induction of the expression of pro-angiogenic factors, e.g., VEGF-A [2,36].

During angiogenesis, new blood vessels develop from the existing endothelial lined vessels, contributing to the degradation of the vascular basement membrane and remodeling of the extracellular matrix (ECM), which is aided by the participation of MMPs. Specific MMPs enhance angiogenesis by helping detach pericytes from vessels undergoing angiogenesis. In addition, MMPs can also contribute negatively to angiogenesis through the generation of endogenous angiogenesis inhibitors [2,29,37,38].

In our study, we observed the co-expression of specific MMPs and TIMPs, namely MMP-9/MMP-2 and TIMP-1/TIMP-2. Regarding MMPs, a slight decrease was observed in the expression of MMP-9, and the level of TIMP-1 was increased; however, lower levels of MMP-2 and TIMP-2 were detected in tumorous tissues compared to adjacent healthy tissue samples. MMPs have been suggested to participate in disruption, tumor neovascularization, and the development of metastases. On the other hand, tissue inhibitors of metalloproteinases (TIMPs) inhibit MMPs’ activity [31,38].

Considering the whole process of angiogenesis in the RCC samples studied, the pathway associated with the actions of MMPs may be due to hsa-miR-15b-5p, hsa-miR-99b-5p, hsa-miR-181a-5p as they exert their role in their interactions as tumor suppressors with MMPs, especially with MMP-9 and MMP-2 (Figure 9). With the formation of a new vascular basement membrane and remodeling of the ECM taking place during angiogenesis in RCC, miRNAs might play a specific role in the degradation of the extracellular matrix, thus inhibiting new vessel formation [2,15,18,29].

Earlier studies have shown that upregulation of specific MMP, such as MMP-14 (MT1-MMP), is associated with poor prognosis in cancer patients. It was also indicated that in aggressive human breast and colon cancer, miR-181a-5p was significantly downregulated, and its levels were inversely correlated with the expression of MMP-14. In clinical specimens, higher MMP-14 expression was observed at the invasive front of tumors where miR-181a-5p was already downregulated relative to adjacent normal cells [26]. We may also assume that similarly to the study by Yiyi [26], miRNAs examined in our study slightly decrease the expression level of MMP-9. The increased level of TIMP-1 inhibits the activity of MMPs, while the low level of TIMP-2 most likely tries to stabilize the activity of MMPs, promoting ahead cell invasion and angiogenesis [2,26,29]. It is also worth noting that all these [12,24] assumptions would be fulfilled in the case of developed metastases of RCC. However, nephrectomy and resection of the tumorous part of the kidney diagnosed with RCC would prevent patients from developing this pathological stage.

In the literature, it was also found that hsa-miR-181a expression is upregulated in gliomas [8,14,15]. In a previous study connected to human colorectal carcinoma (CRC), a high miR-181a expression level was significantly related to the worse survival of colorectal carcinoma patients and was negatively associated with the overall survival of CRC patients with advanced liver metastases [8,26]. All of these findings suggest that the miRNAs examined in this study regulate the process of angiogenesis through their putative targets, which affects the clinical outcome of the patients. This assumption is supported by the statistical correlation observed between miRNAs and the pathological grades of patients in our investigation.

In summary, comparing our results with earlier findings that are already well established, we conclude that the miRNAs examined in our study might have a possible tumor suppressor role. However, the downregulation of studied miRNAs connects them through specific targets to the pathway that initiates angiogenesis in primary kidney cancer tissues.

## 5. Conclusions

Overall, this study was successful in analyzing the expression of hsa-miR-15b-5p, hsa-miR-99b-5p, and hsa-miR-181a-5p. Downregulation of these miRNAs suggests their role in the upregulation of angiogenesis-related targets, thus contributing to epigenetic regulation of the process. A statistically significant negative correlation between the pathological status and the expression of the miRNAs studied suggests a possible role in the pathological process of the development of RCC. Using a comprehensive search for common targets in the three miRNA databases, we generated datasets of potential angiogenesis-associated target genes and specified their potential role in angiogenesis in RCC. We assume this study may help to shed some light on the possible roles of hsa-miR-15b-5p, hsa-miR-99b-5p, and hsa-miR-181a-5p in the mechanism of angiogenesis in RCC. Certainly, further experimental and functional studies of specific miRNAs, focusing on angiogenic targets, are critically needed. Future experiments could also further clarify miRNA-target interactions, for example, the role of TIMPs in the process of angiogenesis.

## Figures and Tables

**Table 1:** Clinicopathological data of RCC patients involved in the study.

Number	Gender	Age	Histology	Grade	TNM	Type of Surgery
1.	female	65	cc. Renocellulare	1	pT1b	Laparoscopic Radical Nephrectomy
2.	male	56	cc. Renocellulare	1	pT1b	Open Radical Nephrectomy
3.	male	73	cc. Renocellulare	2	pT1a	Laparoscopic Renal Resection
4.	female	59	cc. Renocellulare	2	pT1a	Laparoscopic Renal Resection
5.	female	76	cc. Renocellulare	2	pT1a	Laparoscopic Radical Nephrectomy
6.	male	66	cc. Papillare	2	pT1a pNx	Laparoscopic Renal Resection
7.	female	62	cc. Renocellulare	2	pT1a	Laparoscopic Radical Nephrectomy
8.	male	53	cc. Papillare	2	pT1a pNx	Laparoscopic Renal Resection
9.	female	74	cc. Renocellulare	2	pT1a	Open Radical Nephrectomy
10.	male	46	Chromofob cc.	2	pT1b	Laparoscopic Nephrectomy
11.	female	64	cc. Renocellulare	1	pT3a	Laparoscopic Radical Nephrectomy
12.	male	78	cc. Renocellulare	3	pT1b	Laparoscopic Nephrectomy
13.	female	65	cc. Renocellulare	2	pT1a	Laparoscopic Renal Resection
14.	female	65	cc. Renocellulare	1	pT1b	Open Radical Nephrectomy
15.	female	48	cc. Renocellulare	3	pT3a pN1	Laparoscopic Radical Nephrectomy
16.	female	68	cc. Renocellulare	2	pT1a	Laparoscopic Renal Resection
17.	female	71	cc. Renocellulare	3	pT1b	Laparoscopic Nephrectomy
18.	male	51	cc. Renocellulare	2	pT1a	Open Radical Nephrectomy
19.	male	53	cc. Renocellulare	2	pT1b	Laparoscopic Radical Nephrectomy
20.	male	64	Chromofob cc.	2	pT1b	Open Renal Resection

T1a: the tumor is organ localized, size < 4 cm; pT1b: the tumor is organ localized, size: 4–7 cm; pT3a: The tumor reaches the segmental branches of the renal vein, penetrates into the pelvis, the perirenal and/or renal sinus, but does not progress beyond Gerota’s fascia, ccRCC: clear cell renal cell carcinoma, pRCC: papillary type of renal carcinoma. N0/M0: negative lymph node status and no metastases; Nx/Mx: unknown lymph node status; pN1: Micrometastases; or metastases in 1–3 axillary lymph nodes.

## Data Availability

The data presented in this study are available upon request to the corresponding author.

## Supplementary Materials

The following supporting information can be downloaded at https://www.mdpi.com/article/10.3390/biomedicines12071441/s1, Figure S1: Images of the angiogenesis array membranes; Table S1: Protocol of the RT-PCR performed with the Tetro cDNA Synthesis Kit (BIOLINE); Table S2: Results of the Spearman correlation analysis; Table S3A,B: The list of the angiogenesis targets found in databases; Table S4: Sequences of primers used for the real-time qRT-PCR reactions.
