# Supplementary material for: Expression of hsa-miRNA-15b, -99b, -181a and Their Relationship to Angiogenesis in Renal Cell Carcinoma
Source: Biomedicines. 2024 Jun 27;12(7):1441. doi: 10.3390/biomedicines12071441 (PMC11274182; doi:10.3390/biomedicines12071441)
Supplement: Supplementary file 1 [file biomedicines-12-01441-s001.zip › biomedicines-3053634-supplementary.pdf]

Supplementary Materials

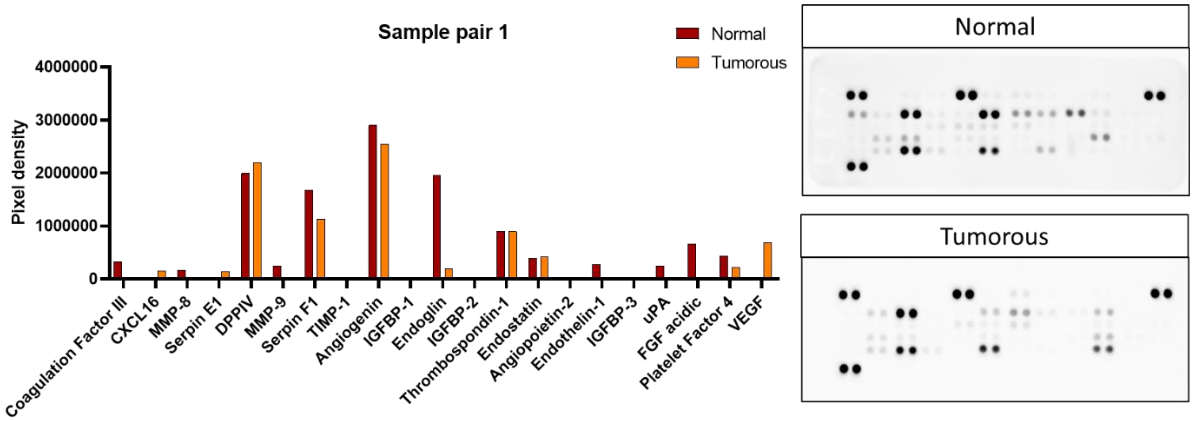

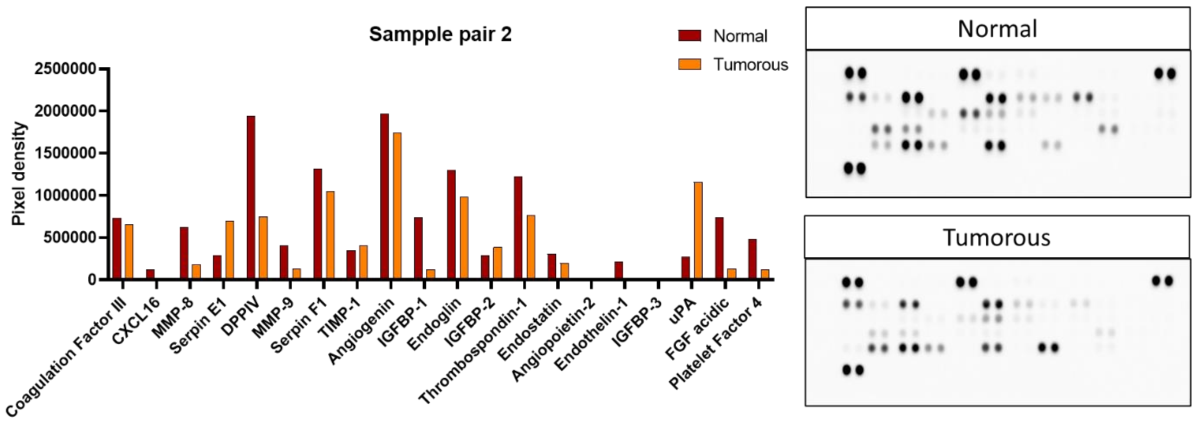

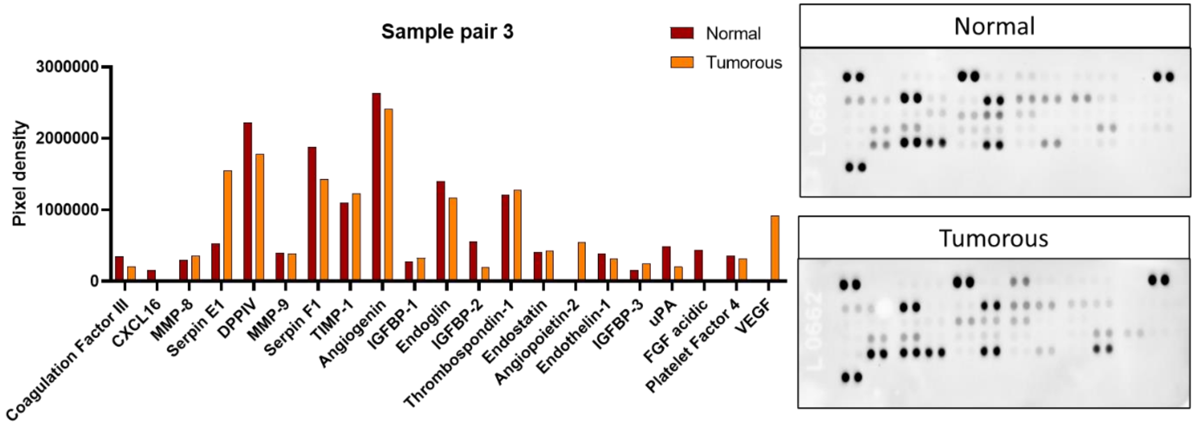
Sample pair 3
Normal
Tumorous
Pixel density
3000000
2000000
1000000
0
Coagulation Factor III
CXCL16
MMP-8
Serpin E1
DPPIV
MMP-9
Serpin F1
TIMP-1
Angiogenin
IGFBP-1
Endoglin
IGFBP-2
Thrombospondin-1
Endostatin
Angiopoietin-2
Endothelin-1
IGFBP-3
uPA
FGF acidic
Platelet Factor 4
VEGF
Normal
Tumorous

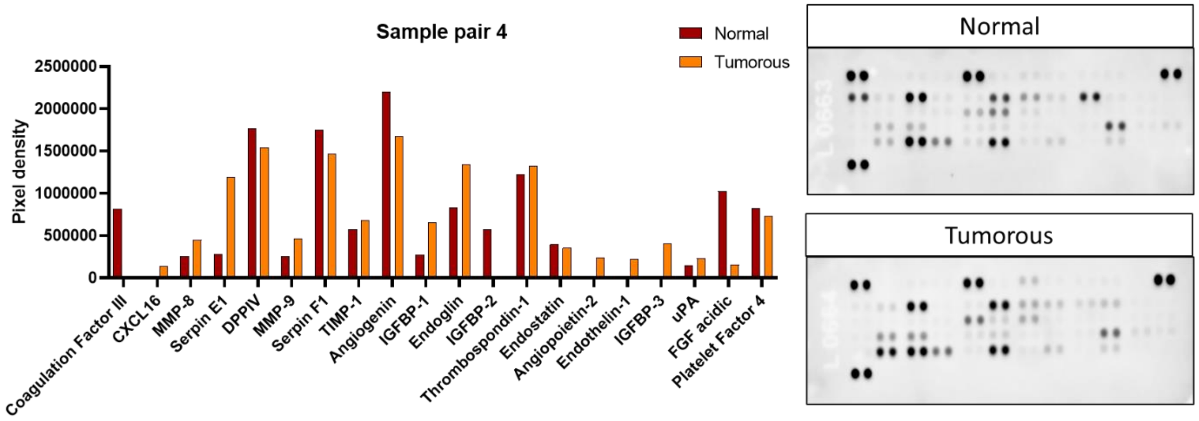
Sample pair 4
Normal
Tumorous
Pixel density
2500000
2000000
1500000
1000000
500000
0
Coagulation Factor III
CXCL16
MMP-8
Serpin E1
DPPIV
MMP-9
Serpin F1
TIMP-1
Angiogenin
IGFBP-1
Endoglin
IGFBP-2
Thrombospondin-1
Endostatin
Angiopoietin-2
Endothelin-1
IGFBP-3
uPA
FGF acidic
Platelet Factor 4
Normal
Tumorous

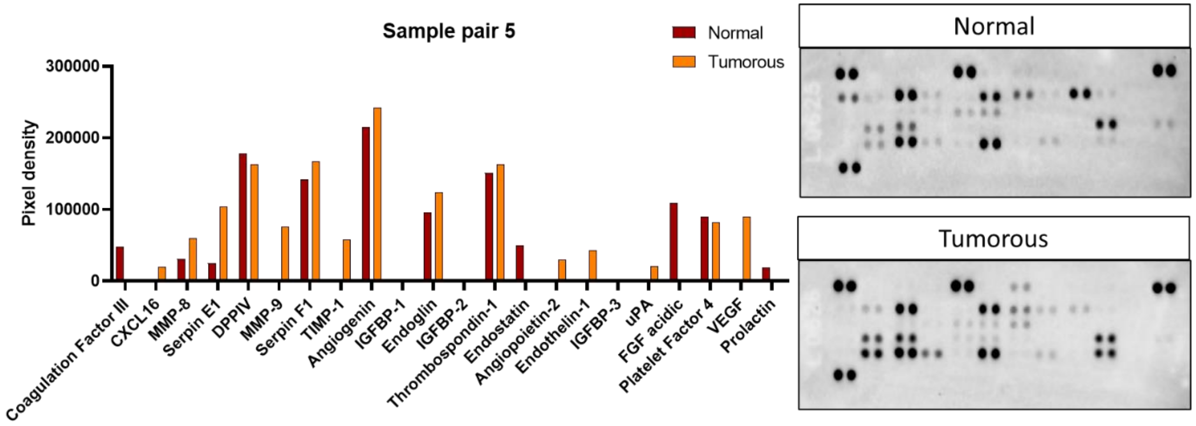
Sample pair 5
Normal
Tumorous
Pixel density
300000
200000
100000
0
Coagulation Factor III
CXCL16
MMP-8
Serpin E1
DPPIV
MMP-9
Serpin F1
TIMP-1
Angiogenin
IGFBP-1
Endoglin
IGFBP-2
Thrombospondin-1
Endostatin
Angiopoietin-2
Endothelin-1
IGFBP-3
uPA
FGF acidic
Platelet Factor 4
VEGF
Prolactin
Normal
Tumorous

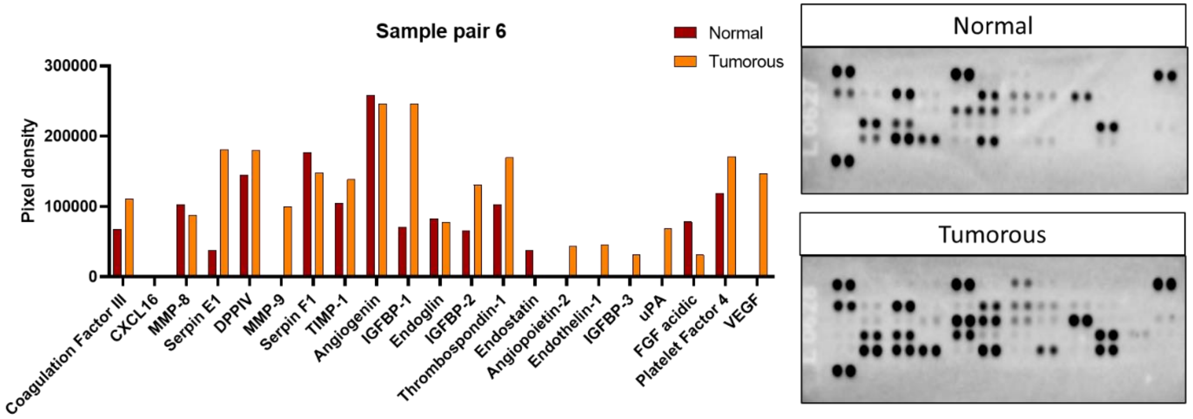
Sample pair 6
Normal
Tumorous
Pixel density
300000
200000
100000
0
Coagulation Factor III
CXCL16
MMP-8
Serpin E1
DPPIV
MMP-9
Serpin F1
TIMP-1
Angiogenin
IGFBP-1
Endoglin
IGFBP-2
Thrombospondin-1
Endostatin
Angiopoietin-2
Endothelin-1
IGFBP-3
uPA
FGF acidic
Platelet Factor 4
VEGF
Normal
Tumorous

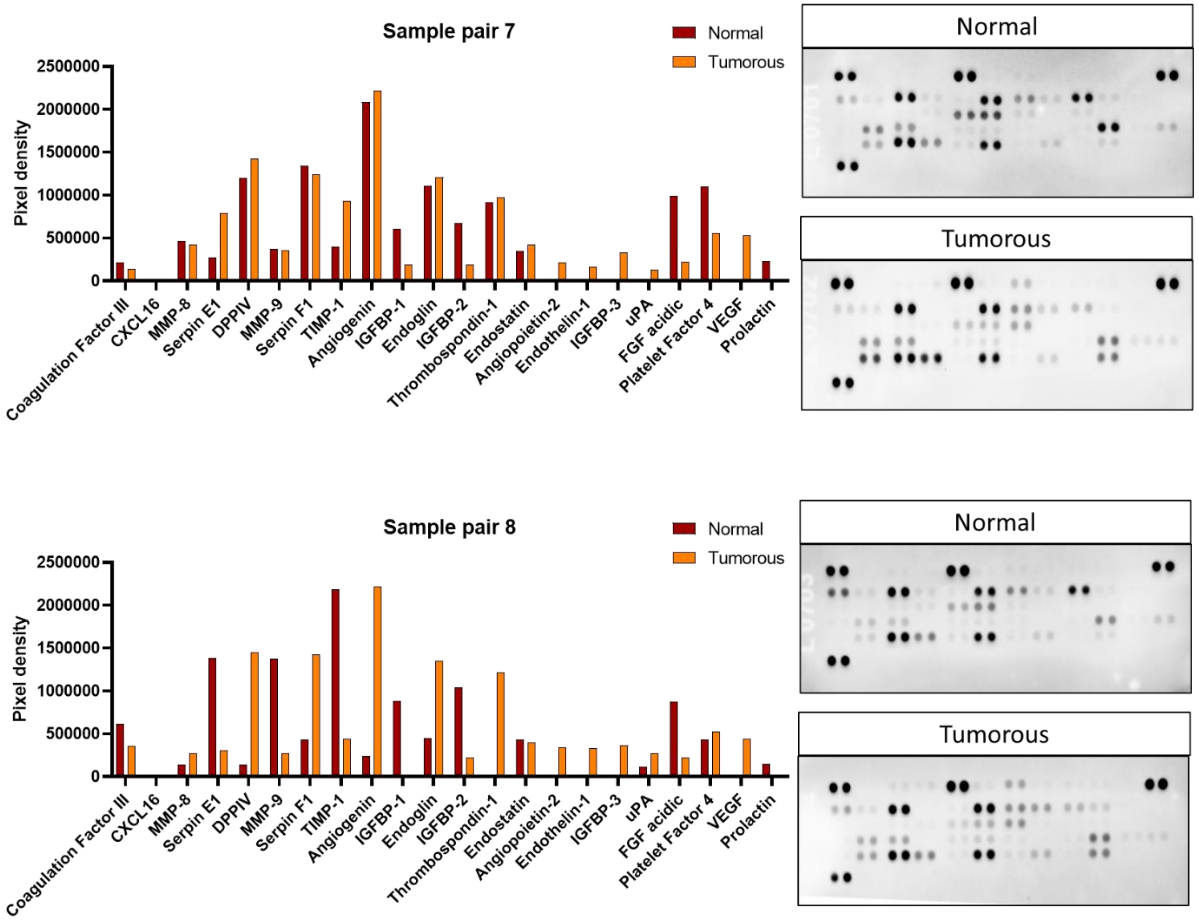

Figure S1. Images of the angiogenesis array membranes

Table S1. Protocol of RT-PCR performed with the Tetro cDNA Synthesis Kit (BIOLINE)

Serial Number	Measured Reagents	Quantities
1.	Total RNA	n µl (for each sample different) *
2.	Primer: Random Hexamer	1 µl
3.	10mM dNTP mix	1 µl
4.	5x RT buffer	4 µl
5.	RiboSafe RNase Inhibitor	1 µl
6.	Tetro Reverse Transcriptase	1 µl
7.	DEPC-treated water	20 - (n+8) µl
Total volume	All measured reagents including RNA	20 µl

Table S2: Results of the Spearman correlation analysis

	has-miR-15b-5p	has-miR-99b-5p	has-miR-181a-5p
**outliers**		**Ø**	
**r**	-0.397	-0.677	-0.479
**95% CI**	-0.686 to 0.001	-0.847 to -0.382	-0.736 to -0.1
**p**	0.0449	0.0001	0.0133
**outliers**		**Q1%**	
**r**	-0.565	-0.794	-0.479
**95% CI**	-0.789 to -0.206	-0.911 to -0.558	-0.736 to -0.1
**p**	0.0033	<0.0001	0.0133
**outliers**		**Q5%**	
**r**	-0.544	-0.794	-0.479
**95% CI**	-0.782 to -0.168	-0.911 to -0.558	-0.736 to -0.1
**p**	0.006	<0.0001	0.0133

Results of the Spearman correlation analysis about the relationship among the relative expression of three types of miRNAs (miR-15b, miR-99b, miR-181a) and the pathological grade of the holding tissue (from 0 to 3). r: the correlation coefficient; CI: the confidence interval; Q: the level of the "aggressiveness" to identify outliers (the permitted maximal chance of identifying false outliers); p: level of statistical significance.

Table S3. A. The targets that can be found in all three databases

Database / miRNA	miRDB, TargetScan, Tarbase common targets
**hsa-miR-15b-5p**	FGF2, RECK, SPRED1, AKT3, PTPN3, HIPK2, VEGFA, MYBL1, PLXNA4, SOCS6, AMOTL1, AMOT, FGF9, RORA, E2F7, LATS2, UNC5B, GLCE, RNF213, FGFR1, ACVR2A,
**hsa-miR-99b-5p**	MBNL1, AGO2, VEGFA, TIMP2
**hsa-miR-181a-5p**	S1PR1, PROX1, ACVR2B, ATP2B1, UBP1, AKT3, IL1A, KLF6, HSP90B1, ETS1, B4GALT1, MYBL1, KDM5A, RORA, RECK, E2F7, PIK3R3, MAP3K2, MBNL1, ITGB8, SERPINE1, SRPK2, TIMP3, HIPK1, MMP14, PTEN, FLT1, PDPK1, SIRT1, SPAG9, EGR3, PIK3C2A,

Table S3. B. The list of the angiogenesis targets found in databases

Database / miRNA	miRDB	TargetScan	Tarbase
**has-miR-15b-5p**	FGF2, ATG14, EPHB2, RECK, SPRED1, APLN, AKT3, TMEM100, PTPN3, HIPK2, VEGFA, MYBL1, PLXNA4, SOCS6, HSPG2, AMOTL1, MEOX2, PCDH17, CLOCK, AMOT, PARVA, EGLN1, NRP2, PDE3B, TARBP2, CDK8, RSPO3, ACVR2B, FGF9, RGMA, RORA, E2F7, DLL4, HPSE2, FGF18, LATS2, SCUBE3, PPM1D, UNC5B, CD40, SAMD4A, GLCE, ADORA2A, PTPRM, EPHA1, VEGFD, PTPN14, NRARP, DCN, CXCR3, EFNB2, CAPN6, PIK3C2A, RNF213, HOXA3, FBXO22, MACC1, IDH3A, PTEN, MMP19, FGFR1, ACVR2A,	FGF2, ATG14, RECK, SPRED1, APLN, AKT3, TMEM100, PTPN3, HIPK2, VEGFA, MYBL1, PLXNA4, SOCS6, HSPG2, AMOTL1, MEOX2, PCDH17, CLOCK, AMOT, EGLN1, NRP2, PDE3B, CDK8, RSPO2, RSPO3, ACVR2A, ACVR2B, FGF9, FGF1, FGF18, RGMA, RORA, E2F7, DLL4, HPSE2, LATS2, SCUBE3, PPM1D, UNC5B, SAMD4A, GLCE, ADORA2A, PTPRM, EPHA1, VEGFA, NRARP, EFNB2, CAPN6, PIK3C2A, RNF213, HOXA3, MACC1, IDH3A, MMP24, FGFR1,	FGF2, RECK, SPRED1, PTPN3, HIPK2, VEGFA, MYBL1, PLXNA1, SOCS6, AMOTL1, ACVR2A, FGF9, LATS2, UNC5B, RNF213, ACVR1B, ADAMTS5, CXCL5, GFRA1, HGF, PDGFA, PDGFC, SERPINB9, TIMP3, AGO1, AKT3, AMOT, B4GALT1, E2F7, RORA, GLCE, FGFR1, S1PR1,
**has-miR-99b-5p**	FZD8, TIMP2, CTDSPL, MBNL1, AGO2, VEGFA,	FZD8, FZD5, VEGFA, CTDSPL, MBNL1, AGO2, FGFR3, TIMP2, RASGRP3,	MBNL1, AGO2, FGFR3, FGF5, VEGFA, ATP2B1, VEGFA, TIMP2, MMP7, MMP9, VCAN, TIMP2,
**has-miR-181a-5p**	S1PR1, PROX1, ACVR2B, ATP2B1, TGFBR1, UBP1, ADGRB3, AKT3, IL1A, KLF6, BCLAF1, HSP90B1, ETS1, IGF2BP2, B4GALT1, MYBL1, KDM5A, RORA, ADAMTS18, DCN, CNOT2, PBX3, TNS1, RASSF8, RECK, AKIRIN2, E2F7, ESM1, DEPTOR, PIK3R3, MAP3K2, METAP2, PSG9, MBNL1, SGK3, ITGB8, SERPINE1, MEF2A, HDAC9, SRPK2, TIMP3, HIPK1, MMP14, VCAN, GREM1, RIN2, VHL, ERO1A, PTEN, PF4V1, TGFBI, FLT1, CACNA2D2, PDPK1, CCN1, ANTXR1, SIRT1, ADM, HK2, ITGA6, HECW2, SPAG9, CLOCK, GPLD1, EGR3, SRSF10, CDK8, NAA15, JCAD, PIK3C2A, PAK4, PLG, PPP3CB, PDGFRA,	S1PR1, PROX1, ACVR2B, ATP2B1, TGFBR1, UBP1, AKT3, IL1A, KLF6, BCLAF1, HSP90B1, ETS1, IGF2BP2, B4GALT1, MYBL1, KDM5A, RORA, ADAMTS18, CNOT2, PBX3, TNS1, RECK, E2F7, ESM1, DEPTOR, PIK3R3, MAP3K2, METAP2, MBNL1, SGK3, ITGB8, SERPINE1, MEF2A, HDAC9, SRPK2, TIMP3, HIPK1, MMP14, VCAN, RIN2, VHL, PTEN, FLT1, CACNA2D2, PDPK1, SIRT1, ITGA6, HECW2, SPAG9, CLOCK, EGR3, SRSF10, CDK8, NAA15, PIK3C2A, PAK4, HGF, PDGFRA,	S1PR1, PROX1, ACVR2B, ATP2B1, UBP1, AKT3, IL1A, KLF6, HSP90B1, ETS1, B4GALT1, MYBL1, KDM5A, RORA, AKIRIN2, E2F7, PIK3R3, MAP3K2, MBNL1, SERPINE1, SRPK2, TIMP2, HIPK1, MMP14, PTEN, TGFBR2, FLT1, PDPK1, SIRT1, ADM, HK2, EGR3, AGO1, AMOT, AMOTL1, ANGPT2, PIK3C2A, PPP3CB, RECK, SPRED1, TIMP3, ITGB8, SPAG9,

Table S4. Sequences of primers used for the real-time qRT-PCR reactions

Primers	Forward	Reverse
**Timp-1**	GGA GAG TGT CTG CGG ATA CTT C	GCA GGT AGT GAT GTG CAA GAG TC
**Timp-2**	ACC CTC TGT GAC TTC ATC GTG C	GGA GAT GTA GCA CGG GAT CAT G
**Angiogenin**	TGG CAA CAA GCG CAG CAT CAA G	GCA AGT GGT GAC CTG GAA AGA AG
**MMP-2**	TAC TGG ATC TAC TCA GCC AGC A	CTT CAG GTA ATA GGC ACC CTT G
**MMP-9**	GCC ACT ACT GTG CCT TTG AGT C	CCC TCA GAG AAT CGC CAG TAC T
**VEGF**	TTG CCT TGC TGC TCT ACC TCC A	GAT GGC AGT AGC TGC GCT GAT A
**HIF-1α**	ATC CAT GTG ACC ATG AGG AAA TG	TCG GCT AGT TAG GGT ACA CTT C
**FGF-1**	ATG GCA CAG TGG ATG GGA CAA G	TAA AAG CCC GTC GGT GTC CAT G
**VEGFR-1**	CTT TTA CCG AAT GCC ACC TC	ACA GCC CCG ACT CCT TAC TT
**VEGFR-2**	ACC CAC CCC CAG AAA TAA AA	CAT ACA CAA CCA GAG AGA CCA CA
**VEGFR-3**	GTC CTT TGG GGT GCT TCT CT	ATC CTT GTG CCG TCT CTC A
